# Proof of concept for voice based MRI scanner control using large language models in real time guided interventions

**DOI:** 10.1038/s41598-025-11290-6

**Published:** 2025-08-25

**Authors:** Hui Chen, Moritz Gutt, Othmar Alexander Belker, Daniel Markus Düx, Frank K. Wacker, Bennet Hensen, Marcel Gutberlet

**Affiliations:** 1 Department of Diagnostic and Interventional Radiology, Hannover Medical School, Hannover, Germany; 2 Research Campus STIMULATE, Otto-von-Guericke University Magdeburg, Magdeburg, Germany

**Keywords:** Medical research, Magnetic resonance imaging, Biomedical engineering

## Abstract

In clinical MRI-guided interventions, the lack of high-quality peripheral equipment and specialized interventional MRI systems often necessitates delegating real-time control of MRI scanners to an assistant. We proposed a voice-based interaction system powered by large language models that enabled hands-free natural language control of MRI scanners. The system leveraged multi-agent collaboration driven by large language models to execute scanner functionalities, including sequence execution, parameter adjustment, and scanner table positioning. In 90 hands-free tests for 18 predefined tasks performed within a real MRI scanning room, the system achieved an overall task completion rate of 93.3% (95% CI 86.2–96.9%). On a consumer laptop without GPU support, the response time for control commands was approximately 5–10.5 s. Our study demonstrates the feasibility of using large language models for voice-based interaction with MRI scanners during interventions, eliminating the need for additional assistants and allowing human-like communication.

## Introduction

Minimally invasive magnetic resonance imaging (MRI) guided interventions offer real-time, radiation-free, high-contrast visualization of soft tissues, allowing precise targeting for procedures such as tumor ablations^1^, biopsies^2^, brachytherapy seed placement^3^, and focal drug delivery^4^. These procedures typically follow a common workflow in open MRI settings involving freehand techniques^5^: trajectory planning, skin entry point localization, and slice alignment for continuous needle visualization during placement.

During real-time needle tracking, the appearance of needle artifacts depends on the material, magnetic field strength, and various sequence parameters, necessitating individual adjustment of imaging settings throughout the intervention^6^. Crucially, such real-time coordination often needs to occur during image acquisition and under sterile conditions, when the interventionalist cannot directly access physical scanner controls. This is typically handled by verbally relaying commands to the technologist in the control room–often requiring the imaging sequence to be paused beforehand. While this workaround allows the procedure to continue, it is inefficient, disrupts the procedural workflow, and increases the likelihood of errors due to miscommunication^7^. Furthermore, it necessitates additional personnel, raising operational costs. Although MRI-compatible communication systems have been proposed to facilitate intra-room dialogue during image acquisition^8^, these do not resolve the core issue of indirect scanner control.

Maintaining MR compatibility and sterility poses significant challenges for the development of direct control devices^9^. Physical alternatives such as sterile-covered Bluetooth mice have shown some feasibility for intra-room scanner control^10^, but are limited by space constraints. Interfaces like sterile dashboards must be manually draped, are fixed in function, and often require users to memorize specific command mappings–posing a barrier to rare or dynamically changing tasks. Additionally, updating these systems typically demands hardware modifications.

The unique working conditions in the operating room and interventional radiology suite have led to the exploration of various concepts of touchless interaction in medical environments, such as eye tracking, gesture and voice control^11^. Compared to the advantages of gesture control in continuously adjusting parameters, voice control was considered more effective in initiating discrete commands^12^. Previous studies have demonstrated the feasibility of voice control in medical environments, including medical image viewer^13^, robotic ultrasound^14^, and interactive hospital room prototype^15^. However, these systems rely primarily on predefined keywords and grammar-based rule sets, requiring users to memorize specific commands, limiting flexibility, and hindering system scalability. Previous work on Autonomous MRI (AMRI) demonstrated a voice-controlled intelligent physical system^16^ for brain imaging^17^, where voice input was used to specify scanning parameters and initiate an autonomous brain screen protocol. While the system enabled automation of a complete imaging workflow, its autonomy, cognizance and adaptability were limited to a predefined task structure, relying on look-up tables and a shallow neural network for slice planning and protocol selection.

With advances in artificial intelligence, large language models (LLMs) have demonstrated human-like problem solving abilities^18^ and enabled more flexible and intuitive voice interactions^18^. Unlike traditional speech recognition, LLM-based voice control prioritizes meaning over exact phrasing, reducing the need for rigid command structures and extensive user training. It has been applied in multiple domains, including virtual assistants for smartphones^19^, physically assistive robots^20^, and mobile robots at the edge^21^, demonstrating its potential for autonomous decision-making and task execution.

This study aimed to explore the feasibility of a contactless voice control system based on LLM in MRI-guided intervention. Specifically, we investigate how such a system can enable direct control of the scanner while maintaining accuracy, safety, and workflow efficiency in the high-noise MRI environment. Our study evaluates the system’s performance in handling predefined tasks, with a particular focus on the impact of external knowledge integration through retrieval-augmented generation (RAG)^22^ and the feasibility of hands-free operation in a real MRI scanner environment.

## Results

We evaluated the control system across 21 basic tasks in three conditions to observe the impact of RAG and voice input on performance (see Table 1). Each task was tested using 25 synonymous commands, with the number determined through preliminary experiments (see Supplementary Tables S1 and S2) to ensure statistical robustness.

Under our experimental settings, the baseline system driven by gpt-4-0125-preview without the RAG and memory modules achieved very stable results on simple tasks that do not require spatial understanding, with task completion rates close to 100% (see Table 1). The RAG module improved the performance of the control system, in particular the task completion rate on the moving slice group task was improved from 8% to 80% ($n=25$, $OR=0.0217$, $P<0.001$), and on the moving table task from 44% to 84% ($n=25$, $OR=0.150$, $P=0.007$), without negatively affecting the performance on other tasks.

With the RAG module enabled, further subdivision tests on the moving slice group task showed a decrease in task completion rate as the number of axes involved in the required displacement increased. When movement was limited to a single axis, a task completion rate of 100% was observed. However, when displacement needed to occur along three axes simultaneously, the completion rate decreased to just 56%. Using the more advanced model gpt-4o-2024-05-13 to drive the multi-agent collaboration system (MACS), the task completion rate improved from 76% to 100% for two-axis movements ($n=25$, $OR=0$, $P=0.02$), with notable improvements from 56% to 84% observed in the three-axis displacement task ($n=25$, $OR=0.242$, $P=0.06$). The average task completion rate across the three subtests increased from 77.3% to 94.7% ($n=75$, $OR=0.192$, $P=0.004$). Similar performance improvements were also observed in the task of moving the scanner table (from 84% to 100%, $n=25$, $OR=0$, $P=0.1$).

When neglecting the accuracy of speech recognition, the best performance was reached by the control system driven by gpt-4o-2024-05-13 and the RAG module enabled with an unweighted average task completion rate of 99.7% on all 21 tasks. Except for the low completion rate (84%, 21 out of 25) for the *“simultaneous movement of the slice group on three axes”* task, a 100% completion rate (25 out of 25) was observed in all other tasks.

The practicality of the system was evaluated in a real scanner room, achieving complete hands-free control of the scanner by activating the RAG and voice modules. With the spelling correction agent, the system achieved a word error rate of 23.6% in speech recognition and an average task completion rate of 93.3% (84 out of 90, 95% CI 86.1% to 97.5%) in 18 tasks. The *“stop sequence”* and *“pause sequence”* tasks were tested amid substantial scanner noise (during active sequences), while other tasks were tested with background noise introduced by the helium compressor when the scanner was idle.

**Table 1:** Task completion rates of multi-agent collaboration system (MACS) on 21 tasks: comparison of the basic version (text input), +retrieval augmented generation (RAG) module (text input), and +voice modules (voice input). User confirmation and the memory module were disabled during this experiment.

		Task completion rate$^\textrm{a}$			
Task (example command)	Related Tools and Perceptrons	base^b^	+RAG^b^	+RAG^c^	+RAG+Voice^c^
Ask the current field of view (FOV) size.	getFieldOfViewRead	$100\%$	$100\%$	/	$100\%$
Set the FOV to 500 mm.	setFieldOfViewRead	$100\%$	$100\%$	/	$100\%$
Expand or reduce the FOV size.	get-, setFieldOfViewRead	$100\%$	$100\%$	/	$100\%$
Display the position of the 1st slice group.	getSlicePositionPcs	$100\%$	$100\%$	/	$100\%$
Set the 1st slice group to (10, -10, 0).	setSlicePositionPcs	$100\%$	$100\%$	/	$100\%$
Move 1st slice group on random patient coordinate system (PCS) axes.	get-, setSlicePositionPcs	${\mathbf {8\%}}^{*}$	${\mathbf {80\%}}^{*}$	/	$80\%$
- on one PCS axis.	get-, setSlicePositionPcs	/	$100\%$	$100\%$	/
- on two PCS axes.	get-, setSlicePositionPcs	/	$76\%^{*}$	$100\%^{*}$	/
- on three PCS axes.	get-, setSlicePositionPcs	/	$56\%$	$84\%$	/
- AVERAGE	get-, setSlicePositionPcs	/	${\mathbf {77.3\%}}^{*}$	${\mathbf {94.7\%}}^{*}$	/
List all templates available for a remote control.	getTemplates	$100\%$	$100\%$	/	$100\%$
Open the template for beat sequence$^{d}$.	openTemplate	$100\%$	$100\%$	/	$80\%$
Save the changes and close the template.	closeTemplate	$100\%$	$100\%$	/	$100\%$
Start one pre-opened sequence.	startSequence	$96\%$	$100\%$	/	$100\%$
Start one non-pre-opened sequence.	get-, openTemplate, startSequence	$100\%$	$100\%$	/	$100\%$
Stop the sequence immediately.	stopSequence	$100\%$	$100\%^{*}$	/	${\mathbf {60\%}}^{*}$
Pause the currently active sequence temporarily.	pauseSequence	$100\%$	$100\%$	/	$100\%$
Restart the paused sequence.	continueSequence	$96\%$	$100\%$	/	$100\%$
Try to gain operational control over the host.	requestControl	$100\%$	$100\%$	/	$100\%$
Reverse the control of the host.	releaseControl	$100\%$	$100\%$	/	$80\%$
Move the table to position 450.	moveTable, *Scanner Table State*	$96\%$	$100\%$	/	$80\%$
Move the table a little out.	moveTable, *Scanner Table State*	${\mathbf {44\%}}^{*}$	${\mathbf {84\%}}^{*}$	${\mathbf {100\%}}$	$100\%$
Check the editing option of the open template.	*Sequence State*	$100\%$	$100\%$	/	/
Show the status of the currently running templates.	*Sequence State*	$100\%$	$100\%$	/	/
Check if the control can be rescheduled.	*Host Control State*	$100\%$	$100\%$	/	/
**Total**		${\mathbf {92.4\%}}$	${\mathbf {98.2\%}}$	${\mathbf {99.7\%}}^{*}$	${\mathbf {93.3\%}}^{*}$

$^{a}$ For each task, *gpt-4-0125-preview* was used to generate 25 semantically similar commands for repeated testing with text input (on base and +RAG version). The commands used in the voice test were 5 randomly selected commands from the 25.

$^{b}$ MACS driven by *gpt-4-0125-preview*.

$^{c}$ MACS driven by *gpt-4o-2024-05-13*.

$^{d}$ The ’beat’ sequence, a product of Siemens, is an MR imaging sequence used to track the needle in real time. Fisher’s exact test: * $P < 0.05$. Untested commands (marked with ‘/’) are explained in the Methods–Evaluation section.

Significant values are in bold.

Table 2 presents the total delay of the voice control system and the delay for each component. The total system delay was observed to be approximately 10.5 seconds, with the majority of the delay attributed to the RAG module. It is important to note that the system control delay was measured for tasks that the agent could complete using only a single tool. For tasks requiring multiple tools sequentially, the number of LLM calls was (n+1). In such cases, the estimated delay for the main control agent can be calculated using the formula: $$(n + 1) \times LLM Runtime per call + Total runtime of all tools$$. In our main control agent, each call to gpt-4o-2024-05-13 took approximately 0.8 seconds.

**Table 2:** Latency caused by different parts of the voice control driven by gpt-4o-2024-05-13 with all module enabled.

Part	Latency / s			
	Sub-part			Total$^{a}$
Voice input module	VAD	NR	STT	
	$<0.1$	$\sim 0.6^{c}$	$\sim 1.3^{\textrm{b,c}}$	$\sim 2.0^\textrm{b,c}$
Multi-agent collaboration	SC-Agent	RAG-Module	MC-Agent	
	$\sim 0.7^\textrm{b,c}$	$\sim 4.2^\textrm{b,c}$	$\sim 1.7^\textrm{b,c}$	$\sim 6.8^\textrm{b,c}$
Voice output module	TTS	/	/	
	$\sim 1.7^\textrm{b,c}$	/	/	$\sim 1.7^\textrm{b,c}$
All				$\sim 10.5^{\mathbf {b,c}}$

VAD, voice activity detection; NR, noise reduction; STT, speech-to-text transcription; SC, spelling correction; RAG, retrieval augmented generation; MC, main control; TTS, text-to-speech synthesis,

^a^Total Latency is the sum of the sub-part latencies and the overhead latency. Overhead refers to the additional time introduced by glue code or system management tasks.

^b^Strongly related to network speed.

^c^Strongly related to audio/text length

A supplementary video is provided to demonstrate the usability of hands-free control in a real scanner room. Using hands-free control, the radiologist successfully started, switched, paused, and stopped MRI sequences, adjusted sequence parameters, and moved the scanner table. To demonstrate the multilingual ability of the voice control system, German input was used in the video, but the language of the responses was set to English. Since noise reduction (NR) and RAG modules were not used, the delay was about 5 s. The video was minimally edited to remove silence when the tester was thinking. In the currently implemented version of the user experience study, during continuous use of voice control, interruption of the explanation of each intermediate action by its next response was allowed to speed up the response process. In addition, the interaction between the user and the voice control system was designed to be half-duplex, which means that the user could interrupt the actions and answers of the master agent at any time, but not the other way around. When the wake-up word is not detected, the system responds: ’I cannot hear you’.

## Discussion

To our knowledge, this study presents the first implementation of an LLM-based voice control system for touchless interaction in the MRI scanner room, utilizing language and contextual understanding rather than predefined rules. While this study does not yet evaluate the clinical outcome directly, the use of LLMs in this context provides a feasible approach to enabling direct scanner control during interventional procedures. By reducing dependence on assistants and rigid command structures, LLMs may enable more autonomous and efficient interventional workflows in future studies.

Previous systems for voice control in the medical field relied on predefined command sets and required user training to ensure precise instruction adherence^12–15,23^, which constraints limited vocabulary flexibility and made spontaneous natural interactions challenging^24,25^. AMRI uses voice input to trigger a predefined brain screening workflow^17^, demonstrating autonomy, cognizance and adaptability within a fixed task structure. In contrast, in this work, the use of LLMs enabled adaptive context-aware command processing without the need for manually crafted heuristics, as demonstrated in various AI-driven voice interaction frameworks^19–21,26,27^.

We describe our voice control system holistically by leveraging the four key characteristics of IPS^16^: Cognizant: The system maps the natural language commands from users to available scanner control tools. Based on the scanner’s current state, it distinguishes between feasible and infeasible operations. The user confirmation mechanism reflects its awareness of capability boundaries. The system actively seeks clarification or determines appropriate parameters autonomously for vague commands. Taskable: The system interprets natural language, possibly vague, commands, planning out and executing concrete actions that are guided by the operational environment determined by three perceptrons. Adaptive: The system learns from and reflects upon experiences and knowledge stored in the memory and RAG modules, enabling behavioral adjustments. Ethical: The user confirmation mechanism ensures system operations remain under user control and enhances transparency and accountability from a regulatory perspective. The wake-up word filter restricts processing to control-related voice commands, minimizing irrelevant data capture risks. Furthermore, data privacy is safeguarded through the implementation of the OpenAI API, which adheres to established privacy standards^28^. The LLM supports multilingual interactions^29,30^, promotes inclusion for diverse linguistic and cultural backgrounds.

Reducing unpredictability in LLM responses remains a challenge. Interactions with LLMs typically focus on a single task with one tool. Providing all documentation at once can overwhelm the agent, increasing the risk of missing critical information, even with the 128k context windows claimed by some LLMs^31^. By retrieving documents dynamically, we increased the prompt’s information density and improved response quality.

The effectiveness of RAG and memory modules varied depending on the complexity of the task. For more complex tasks, such as moving a slice or adjusting the scanner table, these modules played a crucial role in improving performance. They additionally helped to prevent redundant actions, like initiating a sequence which is already activated (see Supplementary Table S6). Although most tasks were eventually completed even with unnecessary additional operations, minimizing the LLM’s runtime reduces task completion time and lowers token costs. In contrast, for simpler tasks, these modules often proved unnecessarily and could even introduce risks, such as deviations from the output format. For example, in 860 tests with RAG enabled, the gpt-4-0125-preview model failed three times to produce the correct structured output, which prevented the execution of specific scanner operations via function calls, while no such errors occurred in 525 tests without RAG ($OR=0$, $P=0.293$). An example of these structured output failures is provided in the supplementary material. Thus, it was prudent to restrict the use of these modules to tasks that clearly benefited from additional context and guidance.

The capabilities of the base LLM impact the usability of our control system. In Table 1, at the time of writing, the most advanced OpenAI model gpt-4o-2024-05-13 had an advantage in tasks where gpt-4-0125-preview performed poorly. This improvement may be partially due to the enhancement of the model’s fundamental intelligence^32^. Accordingly, we anticipate that future model iterations such as GPT-5 could potentially lead to further gains. Additionally, the performance improvement appears to be related to observed changes in the model’s behavior patterns. The gpt-4-0125-preview could not provide textual output while calling a function, meaning that it could only use tools directly without prior reasoning. In contrast, gpt-4o-2024-05-13 offered a rationale when using tools, reflecting an evolution from the simple execution of actions to the ReAct interaction paradigm^33^. Fine-tuning^34^ may be another effective way to improve performance, but we did not try it due to data size limitations.

However, the black-box nature of commercial LLMs prevents a deeper understanding of why specific versions perform better or exhibit different tool-use patterns. This lack of transparency must be acknowledged as a limitation of our current implementation and motivates future exploration of open-source LLMs.

In our early exploration to improve the performance of gpt-4-0125-preview-based MACS on slice movement tasks (see Supplementary Tables S3 and Fig. S1), we demonstrated that the design sophistication of RAG documentation significantly impacts task completion rates. Regardless of the guidance documentation provided, the most frequent failure mode–occurring in approximately 80% of failed attempts–was “moving in the opposite direction.” The other two error types, “moving on the wrong axis” and “moving by the wrong distance,” each occurred in about 20% of failures.

Further subdivision test of slice movement task revealed that completion rates were inversely proportional to the number of axes involved (see Table 1). Even gpt-4o-2024-05-13, which is the most advanced as of now, still only has an 84% completion rate on the three-axis movement task. Slice movement errors resulting from these issues can lead to time-consuming corrections, or even require restarting the setup process. Thus, moving the slice group along one axis at a time is recommended. Similarly, we also hypothesize that simplifying complex instructions into sequential subtasks could improve success rates across various tasks.

Further improvements in task completion rates could be achieved by incorporating a user confirmation loop: after receiving each command, the control agent needs to express its understanding and planned actions. This also helped to increase the user’s control over the entire voice interaction.

Although the task completion rate using voice input (93.3%, 84 out of 90, $n_2=90$) was lower than that of text input (99.7%, 523 out of 525, $n_1=525$), with an odds ratio of 37.4 and $P<0.001$, hands-free operation remained effective in most test scenarios. This was in part attributed to the robustness of the LLM’s command comprehension, as long as the transcription errors did not drastically alter sentence semantics. The biggest challenge facing voice input was the impact of noise on the quality of speech recognition, which was reflected by the low accuracy of 60% (3 out of 5, 95% CI 14.7% - 94.7%) of the *“stop sequence”* task, which was issued in an environment of excessive noise while the imaging sequence was running on the scanner. To clarify the potential risks associated with hands-free usage, we provide a list of possible consequences in the Supplementary Table S7.

Our system architecture can be decomposed into three logical layers:(i) a user-interaction layer that handles voice input and output, (ii) an LLM-based interpretation layer (MACS), and (iii) a vendor-specific execution layer responsible for function calling. Unlike open-source, vendor-neutral frameworks such as Pulseq^35^, Pypulseq^36^, and GammaStar^37^, the current execution layer relies on Siemens-specific Access-I functions. Consequently, porting the system to other scanners would require implementing execution layer around the corresponding vendor APIs, thereby constraining our system’s generalizability and integration potential. We estimate that defining and validating these wrappers in execution layer would demand approximately 1–2 weeks of engineering effort.

In summary, our system provided a scalable intelligent voice solution for contactless MRI control, which could transform MRI-guided interventions by simplifying operations, reducing the need for additional personnel, and minimizing the risk of communication errors. The test commands for the same task were rephrased with different sentence structures and alternative verbs without changing the semantics, which is intended to demonstrate the flexibility of verbal interaction instructions. The human-like interaction and feedback based on an LLM minimized the threshold for users to grasp this control system.

Future research could enhance the system’s cognizance in challenging environments. Specifically, the system should actively evaluate noise to identify situations where its ability to accurately interpret commands is compromised. This awareness of limitations will enable it to autonomously determine when to pause, request clarification, or reject commands, thereby preventing erroneous actions under poor acoustic conditions. Additionally, incorporating speaker identification could help maintain focus on the primary user’s commands in noisy, multi-speaker scenarios.

It should be noted that the current MACS only reports whether its tool invocations succeeded; it cannot yet determine whether those actions actually achieved the user’s intended outcome – that judgment remains the user’s responsibility. Future versions should incorporate self-validation and instant error reporting. Building upon this foundation, further intelligence enhancements are conceivable: For example, MACS could detect during a self-check that an erroneous three-axis slice movement had been executed, roll it back automatically, and recommend breaking the task into separate single-axis moves.

The microphone used for voice recording was not compatible with the MR environment, which limited our testing to a 1.5T MRI scanner (MAGNETOM Aera, Siemens Healthcare GmbH, Erlangen, Germany) to assess the hands-free performance of the system. Although the control worked effectively on the 1.5T scanner, the increased noise levels and stronger magnetic field of the 3T scanner (MAGNETOM Skyra, Siemens Healthcare GmbH, Erlangen, Germany) resulted in significantly degraded audio quality and control failure. To overcome this limitation, future developments should include an MR compatible microphone. The current voice control system involves speech-to-text (STT), LLM and text-to-speech (TTS) stages, resulting in interaction delays. In comparison, OpenAI’s multimodal model pre-demo has demonstrated near real-time interaction capabilities, responding to audio inputs in as little as 232 milliseconds, with an average of 320 milliseconds, comparable to human response times^32^. Exploring the potential of multimodal LLMs that can handle mixed inputs and outputs to reduce the overall system’s response latency is therefore another important area for improvement.

This work is deliberately positioned as a proof-of-concept to demonstrate the technical feasibility of implementing LLM-based voice control for MRI scanners within a real scanning environment. The present study focuses primarily on establishing this foundational capability. Future investigations will build upon this technical groundwork to assess comparative performance metrics–including procedure duration, error rates, user satisfaction, and cognitive load–relative to conventional control paradigms, in which real-time scanner operation is delegated to an assistant. In addition, future studies should systematically investigate the variability in performance across different operators and settings to comprehensively evaluate the system’s robustness. These current limitations and corresponding future work directions are summarized in Supplementary Table S8.

## Methods

This study was prospectively designed and conducted between February and August 2024, focusing on the integration of LLMs into a touchless voice control system for MRI scanners. This section introduces all the necessary hardware and software required for deployment and the statistical methods used to evaluate the usability of the system. The prompt words for each LLM agent were provided in the supplemental material to ensure stable reproduction. All procedures were conducted in accordance with relevant guidelines and regulations, including the Declaration of Helsinki. The voice control tests and supplementary video involved only one of the co-authors, who voluntarily participated and signed a written informed consent form for both participation and the publication of identifiable information, including voice and facial images. As no external participants or sensitive personal data were involved, formal ethical approval was not required.

### Requirement analysis

Hatscher et al. surveyed radiologists’ interactions with MRI during interventions, identifying starting and stopping sequences, and image plane translation as the most frequent functions^9^. In percutaneous MRI-guided liver tumor ablations, as described by Fischbach et al., interventionalists moved patients out of the scanner for surgical preparation and back in for continuous imaging to monitor the needle position, highlighting the need for scanner table control^38^.

Remote control of the MRI scanner was carried out via the commercially available programming interface Access-I (Siemens Healthcare GmbH, Erlangen, Germany). Taking into account the interaction requirements described above, the critical interactions were identified as follows:

- **Controlling sequence progress**
  1. Starting
  2. Stopping
  3. Pausing
  4. Continuing
- **Adjusting sequence parameters**
  5. Field of View (FOV) size
  6. Slice group position
- **Moving the scanner table**
  7. Moving in/out/to a specific location

### Prototype development

**Fig. 1:**
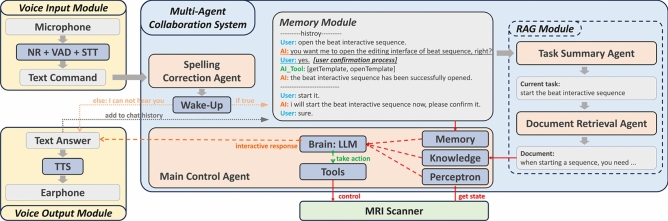
Voice-based touchless control system for MRI scanners. Our voice-controlled system consists of three primary modules: a voice input module for capturing and processing spoken commands, a multi-agent collaboration system (MACS) that interprets and executes commands to control the MRI scanner, and a voice output module for providing auditory feedback. ’Interactive response’ represents three actions – report action result, request confirmation, and request for further explanation.

Figure 1 illustrates our voice control system’s architecture. It included a voice input module that captured sound through a microphone and activated speech-to-text transcription when voice activity was detected. A multi-agent collaboration system then controlled the MRI scanner based on the spelling-corrected results. The main control agent managed communication with the MRI scanner, while its memory module recorded all interaction history, and the perception module obtained the scanner status, including the current sequence, table position, and control permissions through five Access-I query functions. The RAG module included the task summary agent and the document retrieval agent, which analyzed the current task and provided the necessary background knowledge to the main control agent. The wake-up module ensured that only the control commands containing the wake-up word were executed.

It was developed as a web application with distinct front-end and back-end components. The front-end, accessible via modern web browsers (Google Chrome or Microsoft Edge), was responsible for capturing voice streams from the microphone. In addition, although our application could be used completely hands-free, a user interface was built to set user preferences, such as the interaction language and the LLM model to be used. All intermediate results during voice control were visualized in real-time on the front-end via an HTTP-based WebSocket.

For voice activity detection (VAD), we used the browser version of Silero VAD v4 to activate the audio recording only when the interventionalist was speaking. It is a pre-trained, enterprise-grade open-source voice activity detector which processes an audio block (30+ ms) in less than 1 ms on a single CPU thread, demonstrating good performance in different audio domains, background noise levels, and quality conditions^39^. The audio was divided into chunks of 96 ms for the detection of voice activity. The recording continued until more than 1.5 seconds of silence was detected, indicating the longest pause allowed within a single sentence by the interventionalist.

In the back-end, the NR component was implemented before speech recognition. Taking into account the rhythmic background sound of the helium compressor and noise introduced by the running scanner, resembling instruments like drums and bass, we used Demucs *mdx_extra* version^40^ to extract clean vocals, which was originally designed for the separation of music sources. We implemented it on our server and invoked it via an HTTPS-based REST interface. In our evaluation, Demucs took approximately 0.6 seconds to denoise 10 seconds of audio on an NVIDIA L4-24GB GPU (see Table 2).

Once sentence-level speech was obtained, it was processed in the back-end for speech-to-text (STT) recognition. We used OpenAI STT large-v2 Whisper API (OpenAI, 2024, USA) and constructed a coherent context as text prompts (see the supplemental material), incorporating key information such as wake-up keywords, sequence names and operation keywords, to enhance this STT model and improve transcription accuracy^41^. The transcription results are then transferred to the MACS.

After the MACS interpreted user intentions and controlled the MRI scanner functions via the Access-I programming interface, the main agent explained its behavior and reported the execution results to the user’s earphone using the text-to-speech (TTS) component within the voice output module, powered by the OpenAI TTS API (OpenAI, 2024, USA).

### Multi-agent collaboration system

The MACS was designed to interpret and execute interventionalists’ commands to control the MRI scanner. The spelling correction agent was employed to enhance the accuracy of speech recognition by correcting potential errors (e.g., changing the ’bit sequence’ to the ’beat sequence’). The retrieval augmented generation (RAG) technology improves LLMs by retrieving relevant external information to provide more accurate and contextually relevant responses, especially for complex tasks requiring the latest or specific knowledge^22^. In our RAG module, the task summary agent extracted and summarized the current task from historical dialogues, while the document retrieval agent queried the document database for the most relevant guideline to assist the master agent in controlling the scanner. All roles and behaviors of the agent were defined through prompt engineering^42^. The prompt words for all agents are included in the supplementary material, along with a discussion of our approach to designing the RAG document content (see Supplementary Tables S3, S4 and Fig. S1) and the evaluation of our document retrieval agent’s performance (see Supplementary Tables S5).

**Fig. 2:**
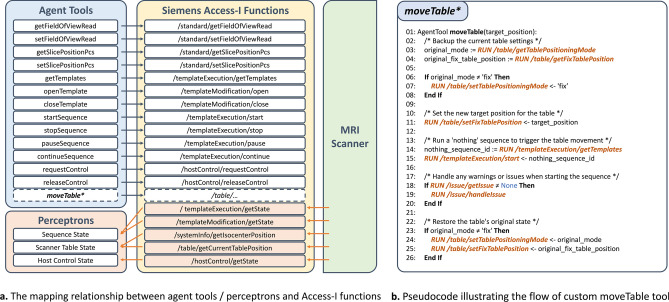
Integration of siemens access-i functions for MRI scanner control. The panel in (**a**) shows how Siemens Access-I functions were integrated as tools and perceptrons to enable the main control agent to manage scanner states and execute MRI scanner functions through function-calling. (**b**) highlights the development of a custom moveTable tool, which was created by combining multiple Access-I functions since Siemens does not provide direct table movement control.

Function-calling^43^ is the ability of LLM to enable effective use of tools and interaction with external APIs. We encapsulated 22 Siemens Access-I functions into 14 external tools and three perceptrons, allowing them to be used by the large language model through function-calling. Each tool came with a clear description to explain its purpose. Thus, the main control agent was equipped to sense the MRI scanner through perceptrons and interact with it using tools corresponding to the commands (see Fig. 2a). Most interactions were directly supported by Access-I functions. However, the table positioning tool was developed by combining multiple Access-I functions, as direct control was not provided. A detailed explanation of this development was shown in Fig. 2b and is discussed in the following section.

### Custom moveTable tool

We combined multiple Access-I functions to create a tool that controlled the scanner table (see Fig. 2b), as the basic library of Access-I functions does not provide a function for direct control of this. Specifically, we took advantage of this feature of the scanner: in the “fix” table positioning mode, if the target table position is set via the “setFixTablePosition” interface, the table will be moved to this position. Therefore, after switching the positioning mode to “fix” and setting the “fixTablePosition” parameter, we trigger the table movement by starting a dummy sequence and then return the positioning mode to its original state.

The scanner table coordinates could be described using either absolute (relative to the scanner) or relative (relative to the current iso-center) coordinates. In our tests (see Supplementary Table S4), there was no significant difference in the completion rate of the “moving table relatively” task between the two position description methods (sample size = 25, completion rate 84% for relative and 76% for absolute coordinates with RAG; $P=0.725$). Therefore, we set the tool to default to relative coordinates but included a convenient switching function between them in the front-end.

### User confirmation process

Considering radiologists’ concerns about the accuracy of STT and the potential risks of LLM illusions, we introduced a user confirmation process to enhance their control over the entire system. When enabled, this process requires the main control agent to explain its intended actions (e.g., calling a tool) to the user before execution, ensuring transparency and safety. The main control agent will not proceed with the action until the user provides explicit approval. An example of this user confirmation process is illustrated within the storage module in Fig. 1. It’s activation and deactivation have been implemented through prompting engineering (see Prompt 1).

**Algorithm 1:**
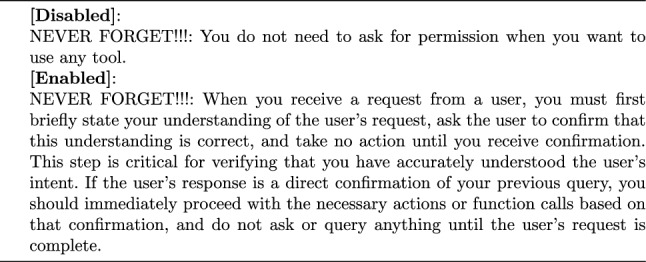
Prompt for user confirmation process.

### LLM-driven feedback and decision explanation behavior

We observed that LLM-based control systems exhibited natural, perception-based feedback and decision explanation behaviors when processing user commands.

For well-defined commands (such as updating the sequence state, querying scanner status or sequence parameters, or adjusting sequence parameters or scanner table position to a specific value), the LLM directly utilized the corresponding tool and reported the outcome. When users issued vague commands (e.g., “move the slice group slightly towards the caudal side”), we identified two distinct behaviors: either the agent autonomously determined the displacement value and performed the movement, or it requested clarification from the user regarding the precise distance before proceeding.

LLM will determine whether the task requires multiple steps to complete based on the current scanner status. It alternated between “using the tool” and “reporting the intermediate result”, ultimately providing a comprehensive summary once the task was completed or failed. Additionally, the LLM rejected unreasonable tasks, such as attempting to move the scanner table beyond its maximum displacement limit.

### Hardware deployment

Our System was deployed on an HP 15-fc0376ng notebook (HP, 2023, purchased in Germany), which interfaced with the scanner’s host computer via an Access-I router. A Sony WH-1000XM4 Bluetooth headset (Sony, 2020, purchased in Germany) was used for microphone input and audio output, connected to the laptop through an ASUS USB-BT500 Bluetooth 5.0 adapter (ASUS, 2020, purchased in Germany) and a USB extension cable, effectively bridging the communication gap caused by the shielding of the scanner room.

### Evaluation

Considering the seven interaction requirements outlined in section Requirement Analysis, 21 basic tasks (see Table 1) were formulated to quantitatively evaluate the performance of the proposed control system under three conditions: without RAG using text input, with RAG using text input and with RAG using voice input. For each task, 25 synonymous commands were generated using the gpt-4-0125-preview model. The choice of 25 commands per task was based on insights from preliminary experiments, which are summarized in Supplementary Tables S1 and S2. To streamline the evaluation, user confirmation was disabled, but it is demonstrated in the supplementary video. Since the tasks were tool-specific, the memory module was also disabled, with its function further discussed in supplemental material. To isolate the performance of the MACS from potential errors in speech recognition by the “Voice Input Module”, all tasks were entered into the system in text form, except when evaluating voice usability.

- Necessity analysis of RAG module: The probability of task completion by MACS, both with and without the RAG module, was first assessed using the Access-I simulator, where commands were input directly as text to simulate an accuracy of 100% for speech recognition.
- Model Comparison: Our MACS was initially powered by gpt-4-0125-preview, which was used for most performance tests. At the time of writing, the latest OpenAI model was gpt-4o-2024-05-13 (OpenAI, 2024, USA). Thus, we evaluated this updated model under the same conditions for tasks where gpt-4-0125-preview had lower completion rates.
- Impact of task complexity on results: To assess how task complexity affected completion rates, the moving slice group task was further divided into uniaxial, biaxial and triaxial motions. For each of these subtasks, gpt-4-0125-preview generated 25 random instructions.
- Hands-free usability via voice modules: The system’s hands-free performance was evaluated on a 1.5T MRI scanner (MAGNETOM Aera, Siemens Healthcare GmbH, Erlangen, Germany), and each task was tested using five random commands. The test was carried out by an interventional physician with two years of experience.

Not all tests were conducted across all system configurations. In Table 1, slash symbols (’/’) indicate tests that were not performed for specific configurations. For the baseline system, after observing very low performance (8%) on general slice movement tasks, subdivision testing was deemed unnecessary. With the newer model (gpt-4o-2024-05-13), testing focused only on tasks where the previous model showed room for improvement. In voice testing, the slice movement task was simply tested using five commands randomly selected from the full test set, without subdividing the test according to the number of axes. It should be noted that these five randomly selected commands consisted of two single-axis movement tasks and three dual-axis movement tasks. The state checking tasks (last three rows) were not tested in voice mode as they provide information that is directly visible on the scanner display so have limited practical utility in voice-control scenarios.

### Statistical methods

In evaluating the RAG and voice modules across 21 tasks, we focused solely on outcomes: e.g. the task of starting an already prepared sequence for needle guidance (’beat’ sequence) was considered to be successfully completed, whether initiated with ’*start*[*beat*]’ or via ’$close[beat] \rightarrow open[beat] \rightarrow start[beat]$’.

We analyzed task completion rates using a two-tailed Fisher’s exact test^44^, with p-values indicating the statistical significance of the associations observed between the compared proportions. Given the small sample size, an alpha level of 0.05 was used and the odds ratio (OR) measured the strength of the associations between groups.

The voice input module was evaluated using the word error rate^45^. Additionally, we measured the response latency for the hands-free control, defined as the interval from the end of user speech to the final response of the main control agent during a single-tool task, accounting for latency from various system components.

## Supplementary Information

Supplementary Information 1.

Supplementary Information 2.

Supplementary Information 3.

## Data Availability

The datasets generated and/or analyzed during the current study are not publicly available due to their storage in our private GitLab repository. To ensure scientific reproducibility while working within practical constraints, we are fully committed to supporting replication efforts by other research teams. Upon reasonable request to the corresponding author, we will provide all necessary code components, detailed implementation guidance, and technical assistance to qualified researchers interested in reproducing or building upon our work. This support includes sharing the complete agent architecture, prompt designs, and integration methods that would enable implementation in other research environments.
