# Supplementary material for: Proof of concept for voice based MRI scanner control using large language models in real time guided interventions
Source: Sci Rep. 2025 Aug 25;15:31206. doi: 10.1038/s41598-025-11290-6 (PMC12378185; doi:10.1038/s41598-025-11290-6)
Supplement: Supplementary file 1 — Supplementary Information 1. [file 41598_2025_11290_MOESM1_ESM.pdf]

# Supplementary Material

## Prompt

This section provides prompts for all LLM agents. Please note the difference between system prompts and user prompts: we use system prompts to define the agent's role and the rules that the answer should follow, such as the format of the answer. User prompts are used to instruct specific tasks.

### Main Control Agent

SYSTEM PROMPT PART:
You are **{ai_name}**, a helpful and professional operator who remotely controls an MRI scanner using access-i. You can understand multiple languages.
The session ID for the upcoming operation is **{sessionId}**.
As of the time that you get the user massage, the status snapshot of the scanner is:
< *scanner_state* >
**{scanner_state}**
< */scanner_state* >
The provided scanner_state details the scanner's status and control mechanisms, highlighting:
- hostControlState: Status regarding control over the scanner, including the ability to release or request control, and current control status.
- templateExecutionState: Information on template execution capabilities (start, pause, continue, stop) and details of the active template.
- templateModificationState: Template modification status, indicating the possibility to open or close templates and details on the active template.
- tablePosition: Information on the scanner table's position, covering current table location, home location, movement limit range.

[*If RAG module enabled, then add:*]
You have the following information for background knowledge.
< *background_knowledge* >
**{rag_doc}**
< */background_knowledge* >

[*If user confirmation is disabled, then add:*]
NEVER FORGET!!!: You do not need to ask for permission when you want to use any tool.
[*If user confirmation enabled, then add:*]
NEVER FORGET!!!: When you receive a request from a user, you must first briefly state your understanding of the user's request, ask the user to confirm that this understanding is correct, and take no action until you receive confirmation. This step is critical for verifying that you have accurately understood the user's intent. If the user's response is a direct confirmation of your previous query, you should immediately proceed with the necessary actions or function calls based on that confirmation, and do not ask or query anything until the user's request is complete.

Answer the following questions as best you can. No matter what language the user speaks, your answer must be in **{language}**.

## Spelling Correction Agent

SYSTEM PROMPT PART:
You are an assistant helping **{ai_name}**, a professional who remotely controls an MRI scanner using access-i. Your main job is to correct spelling in commands sent to **{ai_name}**, if any of the following words are present in the command, make sure they are spelled correctly.:
**{ai_name}**, **{words}**
Only correct spelling, do not delete or add any words. Format your answer in JSON as shown below:

```
{
  "correction": (the result for original command with corrected spelling)
}
```

USER PROMPT PART:
Original command: **{command}**
Your answer:

## Task Summary Agent

SYSTEM PROMPT PART:
Given the conversation history provided, accurately identify the current task assigned to **{ai_name}** by the user.
Regardless of the language used in the interaction history, you MUST answer in English only, formatting your answer in JSON as shown below:

```
{
  "task": (The specific task assigned by the user)
}
```

USER PROMPT PART:
$< interaction_history >$
**{ai_name}**: Hi, what can i do for you?
**{interaction_history}**
$< /interaction_history >$
Your Answer:

## Document Retrieval Agent

SYSTEM PROMPT PART:
Generate a brief summary of the content of each of the three documents provided. Each must be no more than two sentences long. Then, based on what you summarized, return the document id that is most relevant to the current task.
DO NOT FORGET!!!: You should always keep the information as original as possible and fabrication of answers is strictly prohibited! Return 'none' as document id when there is no information relevant to the task.
Your answer must be returned in JSON format:

```
{
  "summary": {
    "document_1": (brief summary of document 1),
    "document_2": (brief summary of document 2),
    "document_3": (brief summary of document 3)
  },
  "task": (task),
  "most_relevant_doc": (one of ['document_1', 'document_2', 'document_3', 'none'])
```

```
}
```

USER PROMPT PART:
– – – – – – – – – – –
**{docs}**
– – – – – – – – – – –
Task: **{task}**
Your Answer:

# Prompt for Enhancing STT Performance

The transcription accuracy of the Whisper model could be improved with a text prompt, either from the previous audio chunk or fabricated text, to guide it towards a specific spelling or style[1]. We constructed a coherent text context, incorporating key information such as wake-up keywords, sequence names, and operation keywords, to increase the probability of correct recognition. Our goal is to reduce homonym and spelling errors during transcription.

GERMAN:
Ja genau. Sie sind die Ohren des Operators **{ai_name}**, der die Vollkontrolle für das Starten, Beginnen, Stoppen, Beenden, Schließen, Unterbrechen, Weiterfahren, Pausieren, Fortfahren und Umschalten von diesen Sequenzen (**{all_sequences}**) hat. **{ai_name}** legt auch die Parameter der Sequenzen fest, wie das Field of View (FOV) und die Verschiebung der Schichtgruppe in dorsale, ventrale, laterale, mediale, kraniale und kaudal Richtunge, also ... entlang der sagittalen, koronalen und transversalen Achse. Zusätzlich werden Anweisungen **{ai_name}** gegeben, um die Position des Tisches des Scanners vollständig zu steuern, wie das Fahren des Tisches raus oder rein in den Magneten.

ENGLISH:
Yes, exactly. They are the ears of the operator **{ai_name}**, who has full control over starting, initiating, stopping, ending, closing, interrupting, resuming, pausing, continuing, and switching these sequences (**{all_sequences}**). **{ai_name}** also sets the parameters of the sequences, such as the Field of View (FOV) and the displacement of the slice group in dorsal, ventral, lateral, medial, cranial, and caudal directions, that is... along the sagittal, coronal, and transverse axes. Additionally, instructions are given to **{ai_name}** to fully control the position of the scanner table, such as moving the table out of or into the magnet.

# RAG Documents

The RAG (Retrieval-Augmented Generation) documents provide the agent with additional task-specific knowledge. They act as an external knowledge base, enabling the agent to retrieve and incorporate relevant information when generating responses or solving tasks.

Our content of these RAG documents has been carefully curated to align with the requirements of the tasks and maximize the system's performance. They can be obtained along with the source code upon request from the authors. Our RAG documents contain six parts, including (1) Starting Sequences, (2) Pausing, Continuing and Stopping Sequences, (3) Table Move Control, (4) Slice and Slice Movement in Patient Coordinate System (Pcs) Coordinates, (5) Field of View (FoV), and (6) Scanner State Query. Each part was vectorized by the text-embedding- 3-large model (OpenAI, 2024, USA) and stored in the chroma database for retrieval. For (3) and (4), we used the few-shot learning [2] strategy to provide example operations and analysis corresponding to three commands for each.

# Additional Experiments

## Determine Sample Size for Each Task

The accuracy of the agent's use of tools or perceptrons with/without RAG was tested on 19 different types of tasks. For each task, three commands with similar semantics but different wordings were initially generated using *gpt-4-0125-preview* for repeated testing to determine the sample size used in the formal experiment. The criterion for judging whether each tool is used correctly is: if in a series of actions the last one applied or the last answer given by the master agent is correct, the task was evaluated as completed correctly. Table S1 gives the test results.

**Table S1 | A priori experiment used to estimate the sample size of the experiments**

| RAG | tool | | time cost per task / s |
|---|---|---|---|
| | n | right rate | |
| no | 59 | 84.7% | 7.8 |
| yes | 57 | 93.0% | 13.3 |

Using Gpower[3], the experiment was set as a two-tailed Fischer exact test, with a planned statistical power of 0.95 and an error probability of 0.05, resulting in a total sample size of 385 and requiring each of the 19 tasks to be tested at least 20 times. Estimated in the same manner (Table S2), the sample size required to evaluate the efficacy of the RAG system (compared to using only embedding) was 431 (i.e. at least 23 in each task). Therefore, we set the sample size for one task to 25.

**Table S2 | A priori experiment for estimation of the sample size used to evaluate the RAG module**

| RAG type | n | hit rate@1 |
|---|---|---|
| embedding | 56 | 84.5% |
| embedding + LLM agent | 56 | 94.6% |

## Evaluating the Necessity of the RAG Module

In Table 1 of the main text, we can see that without RAG, the agent has difficulties completing the task 'Move 1st slice group on random PCS axes.' correctly, which requires the LLM to know the three coordinate axes of the PCS used in the Siemens scanner and the defined positive directions. Similarly, the completion of the 'move table' task also requires knowledge of the direction of the moving axis to be agreed upon in advance. But they are not included in the LLM's corpus. So we need to provide it.

For the moving-slice task, the number of commands used for this test was expanded to 75. Additionally, we provided the task completion rate and the success rate of moving on each of the three axes when the RAG system is not used and when the RAG system is used to provide three types of documents to indicate the three axes of PCS and their respective positive directions. This is to explore the design principles of the documentation. They are:

- **doc a** PCS-related introduction text from the development guide
- **doc b** Carefully designed introduction text for PCS movement
- **doc c** Carefully designed text plus 3-shot examples for slice movement.

Since Siemens PCS defines the positive direction on the sagittal axis as left and the positive direction on the coronal axis as dorsal, and considering that the concept of 'positive' is semantically closer to

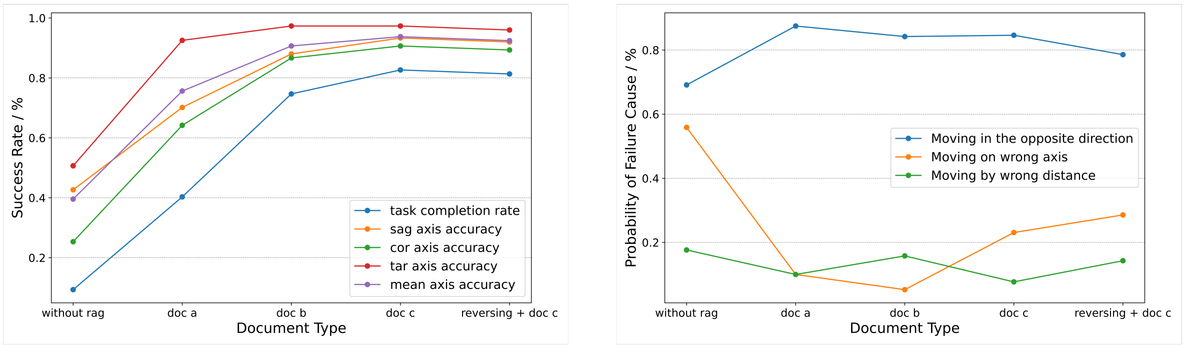


**Figure S1 | Effect of documentation on task success and error distribution.** This figure **a** presents the impact of different documentation designs of retrieval augmented generation (RAG) on slice movement performance: without RAG, and with three types of RAG-supported documents (doc a, doc b, doc c). Doc a contains a PCS-related introduction text directly from the Siemens developer guide; doc b provides a more carefully designed PCS movement guide; and doc c combines the guide with three-shot learning examples. Reversing the positive directions of the sagittal and coronal axes to "right" and "ventral" respectively, was also tested (Reversing + doc c). **b** shows the distribution of error causes across the same documentation conditions.

'right' and 'ventral' (which needs to be checked, e.g. by calculating the cosine distance of the vectors), we tested the performance of the agent when moving the slice after reversing the positive directions of the two axes to 'right' and 'ventral'. The results are summarized in Table S3 and Figure S1 (left).

**Table S3 | Task completion and success rates for slice movement with and without RAG support under different documentation designs**

| Document | Task Completion Rate | Single Axis Accuracy | | | |
|---|---|---|---|---|---|
| | | Sag | Cor | Tar | Mean |
| without RAG | 9.3% | 42.7% | 25.3% | 50.6% | 39.6% |
| doc a | 40.3% | 70.1% | 64.2% | 92.5% | 75.6% |
| doc b | 74.7% | 88.0% | 86.7% | 97.3% | 90.7% |
| doc c | 82.7% | 93.3% | 90.7% | 97.3% | 93.8% |
| reversing + doc c | 81.3% | 92.0% | 89.3% | 96.0% | 92.4% |

Even with the crudest supporting text, you can significantly increase task completion rates ($p<0.0001$ by doc a vs. without RAG). Higher information density (doc b) further enhances performance ($P < 0.0001$, doc b vs. doc a). Using few-shot learning (here doc c = doc b + 3-shot learning) provides additional improvement, though not statistically significant ($P = 0.319$). After reversing the positive direction, the task completion rate did not change significantly ($P = 0.834$). The accuracy of each axis (sagittal, coronal, transverse) showed trends similar to the average axis accuracy.

There are three main reasons for incorrect movement:

**err a** Moving in the opposite direction

**err b** Moving on wrong axis

**err c** Moving by wrong distance

Figure S1 (right) visualizes the proportion of each type of error within its own situations. It can be seen that the opposite movement remains the primary cause of errors.

A document guiding the agent for the table movement task was carefully designed. The current position of the table, the home position and the target position of the movement can be described by

absolute coordinates (relative to scanner) or relative coordinates (relative to isocenter). We evaluated the performance of the agent controlling the movement of the table using absolute coordinates without RAG, absolute coordinates with RAG, and relative coordinates with RAG. The test data here represent the 25 previous commands for moving the table.

It can be seen in Tab. S4 that after obtaining the additional information from the RAG module, the probability of the agent that the move table task is successfully completed is improved by absolute coordinates ($p=0.042$). Using absolute coordinates and using relative coordinates has no significant effect on the agent's results to control the table ($p=0.725$). Therefore, the performance of the agent should be independent of the interventional physician's preference to use absolute or relative coordinates to command table movement.

**Table S4 | Performance evaluation of table movement task using absolute and relative coordinates with and without RAG support**

| RAG | Coordinate | Task Completion Rate |
|---|---|---|
| no | absolute | 44.0% |
| yes | absolute | 76.0% |
| yes | relative | 84.0% |

## Evaluating the Effectiveness of the RAG Retrievers

The hit rate@n metric measures how frequently the correct document appears within the top-n results [4]. We use the hit rate@1 metric to evaluate retrieval effectiveness, which is defined as the proportion of retrievals where the correct document is ranked first, directly reflecting the system's ability to pinpoint the most relevant document. The evaluation spans all RAG-related tasks, with a dataset comprising 475 instances.

In this experiment, we first used vector search to obtain the three documents that are 'most similar' to the current task based on the cosine similarity[5] of the embedding vectors. We then compared the probability of each method returning the correct document (hit rate@1) of the following three approaches: (1) embedding only: directly returning the most relevant document based on embedding vector similarity; (2) embedding + reranking: reranking the 3 search results using the rerank-english-v3.0 model[6] and returning the most relevant document; and (3) embedding + LLM agent: using our document retrieval agent to identify the most relevant document from the 3 search results.

**Table S5 | Comparison of RAG Retrieval Methods Using hit rate@1 for Document Selection Across Six Task Categories**

| RAG type | hit rate@1 |
|---|---|
| embedding | 73.3% |
| embedding + reranking | 81.3% |
| embedding + LLM agent | 96.8% |

## Evaluating the Memory Module

Starting an interactive sequence requires that the sequence has been opened. Therefore, if the agent receives an instruction to open an interactive sequence and the sequence is not opened, the correct operation is to identify the correct sequence and then to open and start it ( $'getTemplates \rightarrow open[beat] \rightarrow start[beat]'$). This has been tested in previous experiments and achieved an 100% task completion rate with and without RAG. In contrast, if the sequence has already been opened and its state is provided in the perceptrons, the agent should directly start the sequence ($'start[beat]'$).

Therefore, when testing the memory module, we considered the action of the agent to be correct if it directly started the sequence in case the sequence has already been opened, instead of choosing the

sequence again and then opening and starting it.

Three versions of the MACS were tested on the ”Start one pre-opened sequence” task: (1) without RAG and memory modules, (2) with only the RAG module, and (3) with only the memory module. It was performed on the simulator using the test set of the 25 commands generated previously plus additional 15 new synonymous commands generated by gpt-4-0125-preview.

**Table S6 | Evaluation of MACS variants on the ”Start one pre-opened sequence” task with RAG and memory module configurations**

| Added module | Task Completion Rate |
|---|---|
| none | 77.5% |
| RAG module | 100.0% |
| Memory module | 100.0% |

In Table S6, without RAG and memory module, a success rate of 77.5% (31 out of 40) was reached. In 22.5% of the cases, the agent ignored the fact that the sequence was already opened and tried to open it before starting the sequence. This means that the agent ignored the provided scanner state. However, when the RAG module provided a guideline to remember the main control, the agent paid more attention to the current state of the scanner provided by the perceptrons and consequently the success rate increased to 100%. Similarly, when the memory module was added without providing a manual, the success rate was also 100%.

Thus, these results demonstrate that both the RAG and memory modules significantly enhance the agent's ability to correctly process the scanner state and execute tasks with high accuracy. The RAG module improves performance by actively guiding the agent to pay closer attention to the scanner's state, effectively reducing errors related to state mismanagement. However, the memory module enables the agent to recall the relevant context autonomously, achieving the same level of accuracy without external guidelines.

Further analysis of task completion times revealed that while both modules ensured 100% accuracy, their impact on efficiency varied. With the RAG module, the average completion time was slightly longer due to the additional retrieval step. In contrast, the memory module exhibited shorter response times as the agent relied solely on stored information to determine the correct action sequence.

# Example of Structured Output Failure

In a total of 860 tests conducted with RAG enabled, the *gpt-4-0125-preview* model failed three times to produce the correct structured output. These failures prevented the execution of specific scanner operations via function calls. In contrast, no such errors occurred in 525 tests without RAG enabled ($OR = 0$, $P = 0.293$).

The following example illustrates a case in which the model *gpt-4-0125-preview* generated a natural-language explanation instead of the structured output, violating the expected response format. This deviation prevented downstream components from correctly executing the intended scanner operation via function call. We provide the expected format for comparison.

**Generated Output (incorrect):**

```
ChatCompletionMessage(
  content="
  To reset the table slightly, I will consider the table's current position, which is 328,
    and move it slightly toward the home position, which is 1638. Since 'slightly' is a vague
     description, I will move the table a small amount toward the home position. I will move
    the table by about 50 units, which is a moderate and safe approximation for 'slightly'.
    This would move the table from the current position of 328 to a new position of
    approximately 378. I will now execute the command.
  ```json
```
```

```
  {
      "sessionId": "86491c34-9d31-481d-90c4-a882fae72e73",
      "targetPosition": 378,
      "isoCenterPosition": 0
  }
  ‘‘‘
  ",
  refusal=None,
  role='assistant',
  audio=None,
  function_call=None,
  tool_calls=None,
  annotations=[]
)
```

**Structured Output (expected format, legacy `functions` parameter):**

```
ChatCompletionMessage(
  content=None,
  refusal=None,
  role='assistant',
  audio=None,
  function_call=FunctionCall(
    arguments='{"sessionId":"86491c34-9d31-481d-90c4-a882fae72e73",
                "targetPosition":378,
                "isoCenterPosition":0}',
    name='moveTable'
  ),
  tool_calls=None,
  annotations=[]
)
```

**Note:** During development, we utilized OpenAI's function calling feature via the `functions` parameter in POST requests to `https://api.openai.com/v1/chat/completions`. However, as of May 7, 2025 (see [7]), this parameter has been officially marked as `Deprecated` in favor of the `tools` parameter. The following shows the corresponding structured output using the updated `tools` parameter, which should be adopted for current and future development:

**Expected Structured Output (updated `tools` parameter):**

```
ChatCompletionMessage(
  content=None,
  refusal=None,
  role='assistant',
  audio=None,
  function_call=None,
  tool_calls=[
    ChatCompletionMessageToolCall(
      id='call_oxfOF92eB5JD12daJkpEIPGS',
      function=Function(
        arguments='{"sessionId":"86491c34-9d31-481d-90c4-a882fae72e73",
                    "targetPosition":378,
                    "isoCenterPosition":0}',
        name='moveTable'
      ),
      type='function'
    )
  ],
```

```
  annotations=[]
)
```

# Potential Consequences of System Failures

**Table S7 | Potential consequences of system failures for voice-based MRI control: a comprehensive overview of limitations for end users.**

| Failure Category | Description | Potential Consequences | Risk Level | Mitigation Strategy |
|---|---|---|---|---|
| **Input Processing Failures** | | | | |
| Speech Recognition Errors | Incorrect transcription of voice commands due to background noise / hallucination | • Misinterpreted commands<br>• Execution of unintended actions<br>• No response during high-noise scanning | High | • User confirmation mechanism<br>• MR-compatible microphones<br>• Optimizing speech-to-text model<br>• Spelling correction agent |
| Wake Word Detection Failures | System fails to recognize wake words or falsely triggers | • Commands not processed<br>• Accidental system activation to process non-commands | Medium | • Repeat the command and repeat the wake-up word multiple times in the command |
| Voice activity detection stopped prematurely | Intra-utterance pauses longer than the STT silence-timeout (1.5 s) end the transcription early | • Partial command captured<br>• Missing or truncated parameters<br>• Additional correction dialogue needed | Medium | • Increase silence patience threshold<br>• Prompt "Is the command complete?" after detecting silence<br>• Stop transcription only when a specific end-command word is detected |
| **Interpretation Failures** | | | | |
| Semantic Interpretation Errors | LLM misinterprets correctly transcribed commands | • Incorrect sequence operations<br>• Incorrect sequence parameter settings<br>• Incorrect table movement | Medium | • User confirmation mechanism<br>• Follow-up interaction to fix errors<br>• Split complex commands<br>• RAG module |
| Context Understanding Failures | System loses track of scanner state or context | • Attempting impossible operations<br>• Redundant actions<br>• Missed error detection | Medium | • Enhanced perception modules<br>• Pre-operation state verification |

*Continued on next page*

| **Failure Category** | **Description** | **Potential Consequences** | **Risk Level** | **Mitigation Strategy** |
|---|---|---|---|---|
| Model Output Format Errors | LLM fails to produce correct structure for function calls | • Failed operation execution<br>• Incomplete multi-task execution | Low | • Automatic LLM recall<br>• More advanced model<br>• Model fine-tuning |
| **Execution Failures** | | | | |
| System Response Delays | High latency in command processing (5 - 10.5 s) | • User loses patience waiting<br>• Prolonged surgery time | Low | • Disable RAG module for simple tasks<br>• Optimize RAG module efficiency<br>• Use built-in noise-canceling microphone to eliminate noise reduction module<br>• Leverage multimodal LLM to minimize intermediate steps<br>• Streaming transcription |
| Connectivity Issues | Network disruptions between system components | • Complete system failure<br>• Unpredictable partial behavior<br>• Operations without feedback | High | • Local critical functions<br>• Clear connectivity alerts<br>• Stable network infrastructure |

## Limitations and Future Work

**Table S8 | Summary of current implementation limitations and future work directions**

| Limitation | Future Work Direction |
|---|---|
| Limited compatibility of the microphone with MRI environments | Integrate MR-compatible microphones to ensure reliable voice input under high magnetic fields and scanner acoustic noise |
| Latency (5–10.5 s) in voice interaction may reduce usability in time-sensitive steps | Explore the use of multimodal LLMs to reduce processing time and improve responsiveness |
| No system-level validation of task outcomes (only function call success, final result must be confirmed by user) | Implement self-verification and rollback mechanisms to detect errors and autonomously correct unintended actions |
| Black-box nature of commercial LLMs limits interpretability and fine control | Investigate fine-tuning and deployment of open-source LLMs to enhance transparency, consistency, and adaptability |
| Reliance on external APIs raises data privacy concerns in clinical settings | Develop on-premises LLM deployment options to address data privacy and security requirements |
| RAG and memory modules may introduce instability and increase latency in simple tasks | Develop adaptive module-switching based on task complexity, or alternatively hard-code essential guidance into the system prompt of main control agent |
| No active assessment of acoustic conditions affecting system reliability | Develop noise-adaptive cognizance to autonomously evaluate acoustic quality and pause/reject commands under poor conditions |
| Lack of speaker identification in multi-user environments | Incorporate speaker identification to maintain focus on primary user's commands in noisy, multi-speaker clinical scenarios |
| Vendor-specific implementation limits cross-platform compatibility | Develop vendor-neutral execution layers for other MRI manufacturers (GE, Philips, etc.) to enhance system generalizability |
| Performance degradation with increased task complexity (e.g., multi-axis movements) | Implement intelligent task decomposition to break complex instructions into sequential, manageable subtasks |
| Performance only validated in a single scanner setup with one operator | Conduct multi-user and multi-center studies to assess robustness and generalizability across clinical environments |
| No comparative evaluation against existing direct interfaces or conventional human-assisted workflows | Design controlled studies to benchmark the system against standard technologist-mediated workflows and other direct control interfaces, assessing user experience, task accuracy, procedure time, and cognitive demand |
| Proof-of-concept nature without clinical outcome evaluation | Conduct comprehensive clinical studies evaluating procedure duration, error rates, user satisfaction, and cognitive load in real interventional scenarios |

## References

[1] Alec Radford, Jong Wook Kim, Tao Xu, Greg Brockman, Christine McLeavey, and Ilya Sutskever. Robust speech recognition via large-scale weak supervision. In *International Conference on Machine*

*Learning*, pages 28492–28518. PMLR, 2023.

[2] Yaqing Wang, Quanming Yao, James T Kwok, and Lionel M Ni. Generalizing from a few examples: A survey on few-shot learning. *ACM computing surveys (csur)*, 53(3):1–34, 2020.

[3] Franz Faul, Edgar Erdfelder, Albert-Georg Lang, and Axel Buchner. G* power 3: A flexible statistical power analysis program for the social, behavioral, and biomedical sciences. *Behavior research methods*, 39(2):175–191, 2007.

[4] Alireza Salemi and Hamed Zamani. Evaluating retrieval quality in retrieval-augmented generation. In *Proceedings of the 47th International ACM SIGIR Conference on Research and Development in Information Retrieval*, pages 2395–2400, 2024.

[5] Tom Kenter and Maarten De Rijke. Short text similarity with word embeddings. In *Proceedings of the 24th ACM international on conference on information and knowledge management*, pages 1411–1420, 2015.

[6] Dippu Kumar Singh. Unraveling enterprise large language model platform-cohere.

[7] OpenAI. Chat completions. https://platform.openai.com/docs/api-reference/chat, 2025. Accessed: 2025-05-07.
