# Supplementary material for: Proof of concept for voice based MRI scanner control using large language models in real time guided interventions
Source: Sci Rep. 2025 Aug 25;15:31206. doi: 10.1038/s41598-025-11290-6 (PMC12378185; doi:10.1038/s41598-025-11290-6)
Supplement: Supplementary file 2 — Supplementary Information 2. [file 41598_2025_11290_MOESM2_ESM.pdf]

Supplementary Video S1 | Demonstration of Hands-Free MRI Scanner Control Using Voice Commands

This video demonstrates the usability of hands-free, voice-based control in a real MRI scanner room. It showcases a radiologist performing typical scanning tasks using a large language model-based interface without any physical interaction.

The demonstrated functionalities include:

- [00:00:00 - 00:01:48] Starting, switching, pausing, and stopping MRI sequences
- [00:00:55 - 00:02:08] Adjusting sequence parameters via voice commands
- [00:02:08 - 00:03:02] Moving the scanner table

To highlight the multilingual capabilities of the system, German voice input was used throughout the video, while the system responses were configured to be in English.

Additional implementation details:

- At the beginning of the demonstration, the user confirmation flow was disabled to streamline the interaction. However, it was enabled during the second table movement to demonstrate its function.
- Our noise reduction (NR) and retrieval-augmented generation (RAG) modules were not enabled during this demonstration. The observed audio clarity relied on the built-in noise suppression of the Sony WH-1000XM4 Bluetooth headset (Sony, 2020, purchased in Germany).
- Due to the absence of NR and RAG in this video, the average system response latency was approximately 5 seconds.
- The video was lightly edited to remove periods of silence when the user was thinking.
- The system operates in a half-duplex interaction mode: the user may interrupt the agent at any time, but not vice versa.
- During continuous use, intermediate responses from the system can be preempted by new response to reduce turnaround time.
- When the wake-up word is not detected, the system responds: *"I cannot hear you"*.
